# Multimorbidity and health-related quality of life amongst Indigenous Australians: A longitudinal analysis

**DOI:** 10.1007/s11136-023-03500-3

**Published:** 2023-08-16

**Authors:** Syed Afroz Keramat, Francisco Perales, Khorshed Alam, Rumana Rashid, Rezwanul Haque, Nahid Monasi, Rubayyat Hashmi, Farzana Siddika, Zubayer Hassan Siddiqui, Mohammad Afshar Ali, Natnael Demeke Gebremariam, Srinivas Kondalsamy-Chennakesavan

**Affiliations:** 1 Faculty of Medicine, Centre for Health Services Research, The University of Queensland, Brisbane, QLD Australia; 2 Economics Discipline, Khulna University, Khulna, 9208 Bangladesh; 3 School of Social Science, The University of Queensland, Michie Building (#9), St Lucia, Brisbane, QLD 4067 Australia; 4 School of Business and Centre for Health Research, University of Southern Queensland, Toowoomba, QLD 4350 Australia; 5 Department of Mathematics and Natural Sciences, BRAC University, Dhaka, Bangladesh; 6 Department of Business Administration, Bangladesh University of Professionals, Dhaka, Bangladesh; 7 Department of Economic History, Lund University, Lund, Sweden; 8 Rural Clinical School, Faculty of Medicine, The University of Queensland, 152 West St, South Toowoomba, QLD 4350 Australia

**Keywords:** Chronic condition, Quality of life, Indigenous Australians, Multimorbidity

## Abstract

**Background:**

The burden of multimorbidity has been observed worldwide and it has significant consequences on health outcomes. In Australia, health-related quality of life (HRQoL) is comparatively low amongst Aboriginal and/or Torres Strait Islanders, yet no studies have examined the effect of multimorbidity on HRQoL within this at-risk population. This study seeks to fill that gap by employing a longitudinal research design.

**Methods:**

Longitudinal data were derived from three waves (9, 13, and 17) of the household, income and labour dynamics in Australia (HILDA) Survey. A total of 1007 person-year observations from 592 Aboriginal and/or Torres Strait Islander individuals aged 15 years and above were included. HRQoL was captured using the 36-item Short-Form Health Survey (SF-36), and multimorbidity was defined using self-reports of having been diagnosed with two or more chronic health conditions. Symmetric fixed-effects linear regression models were used to assess how intraindividual changes in multimorbidity were associated with intraindividual changes in HRQoL.

**Results:**

Approximately 21% of Indigenous Australians were classified as experiencing multimorbidity. Respondents had statistically significantly lower HRQoL on the SF-36 sub-scales, summary measures, and health-utility index in those observations in which they experienced multimorbidity. Among others, multimorbidity was associated with lower scores on the SF-36 physical-component scale (β =  − 6.527; Standard Error [SE] = 1.579), mental-component scale (β =  − 3.765; SE = 1.590) and short-form six-dimension utility index (β =  − 0.075; SE = 0.017).

**Conclusion:**

This study demonstrates that having multiple chronic conditions is statistically significantly associated with lower HRQoL amongst Indigenous Australians. These findings suggest that comprehensive and culturally sensitive health strategies addressing the complex needs of individuals with multimorbidity should be implemented to improve the HRQoL of Indigenous Australians.

**Supplementary Information:**

The online version contains supplementary material available at 10.1007/s11136-023-03500-3.

## Introduction

Multimorbidity is a leading public-health burden and a source of significant challenges to healthcare management. In the literature, the term ‘multimorbidity’ is used interchangeably with the term ‘comorbidity’ and can be defined as the presence of two or more chronic conditions within an individual at the same time [1]. These co-occurring diseases may or may not be connected by a causal relationship. In Australia, multimorbidity impacts almost one-fifth of the population and 80% of those over 65 years of age [2, 3].

Australia bears a severe burden from chronic conditions, with 90% of all deaths and major disabilities attributable to single or multiple long-term conditions [4]. The growing prevalence of multimorbidity negatively contributes to health-related quality of life (HRQoL), which refers to individuals’ overall physical health and well-being throughout their lifespan [1]. The potential relationship between multimorbidity and HRQoL has received substantial academic attention over the last decade [5–7]. This body of evidence has revealed that a person’s life can be significantly affected by multimorbidity, including through physical-functioning limitations, psychological problems, and financial burdens [8–10], ultimately reducing their quality of life [11, 12]. Indeed, numerous studies from different settings have empirically confirmed that individuals with multimorbidity tend to have poorer quality-of-life outcomes [9, 12–14], including loneliness, social isolation, stress, anxiety and low life satisfaction [15–17]. This reality applies also to Australia, the country in which the present study is based [1, 2, 12, 18].

In Australia, there are important ethnic-based health disparities structured around Indigenous status, with Indigenous Australians experiencing a disproportionately high disease burden [2, 18]. For instance, Indigenous Australians have a 4.6-fold greater age-standardised burden of cardiovascular disease than non-Indigenous individuals, and diabetes rates are also disproportionately high [19]. This is consistent with the finding that, amongst Indigenous Australians, individuals of all age groups face impediments to accessing health care [20, 21]. Patterns of multimorbidity also vary greatly by Indigenous status, with its prevalence being higher amongst Indigenous (24.2%) than non-Indigenous (20.7%) Australians [22]. In fact, the higher prevalence of chronic condition among Indigenous Australians is a major contributor to the observed disparities in health outcomes between Indigenous and non-Indigenous Australians [19]. A recent study also revealed that multimorbidity is associated with increased healthcare service utilisation, decreased productivity, and diminished perceived health outcomes among Indigenous adults [23]. Taken together, this body of evidence suggests that Indigenous Australians may be particularly vulnerable to multimorbidity and its deleterious effects.

To the best of our knowledge, however, no studies have investigated the relationships between multimorbidity and HRQoL amongst Indigenous Australians. In fact, only a few longitudinal studies have examined the relationship between multimorbidity and HRQoL. These are important research gaps, given the inverse associations between multimorbidity and HRQoL found in cross-sectional studies, as well as the higher incidence of multimorbidity amongst Indigenous Australians. To address these, the present study examines the relationship between multimorbidity and HRQoL amongst Indigenous Australians using a longitudinal research design based on fixed effects models.

## Methods

### Data source and sample selection

The Household, Income and Labour Dynamics in Australia (HILDA) Survey, a major household-based panel survey, was used as the basis for the current study. The HILDA Survey is an annual nationwide representative longitudinal survey initiated in 2001 that collects data on family relationships, health, wealth, income, labour market, employment and education through a combination of face-to-face interviews and self-completion questionnaires [24]. The HILDA project received approval from the University of Melbourne Human Research Ethics Committee.

The HILDA Survey features a representative sample of Australian households residing in private dwellings, identified through a multi-stage sampling approach. The initial survey wave collected data from 13,000 Australian adults from more than 7000 households (household response rate of 66 per cent), and new respondents have joined the sample by marrying or beginning to live with existing sample members [25, 26]. Furthermore, booster samples have been added to the survey since 2011 to maintain representativeness. Wave-on-wave retention rates in HILDA Survey is approximately 96% and are therefore high for international standards [26]. Further details on the sampling procedure can be found elsewhere [27].

**Fig. 1:**
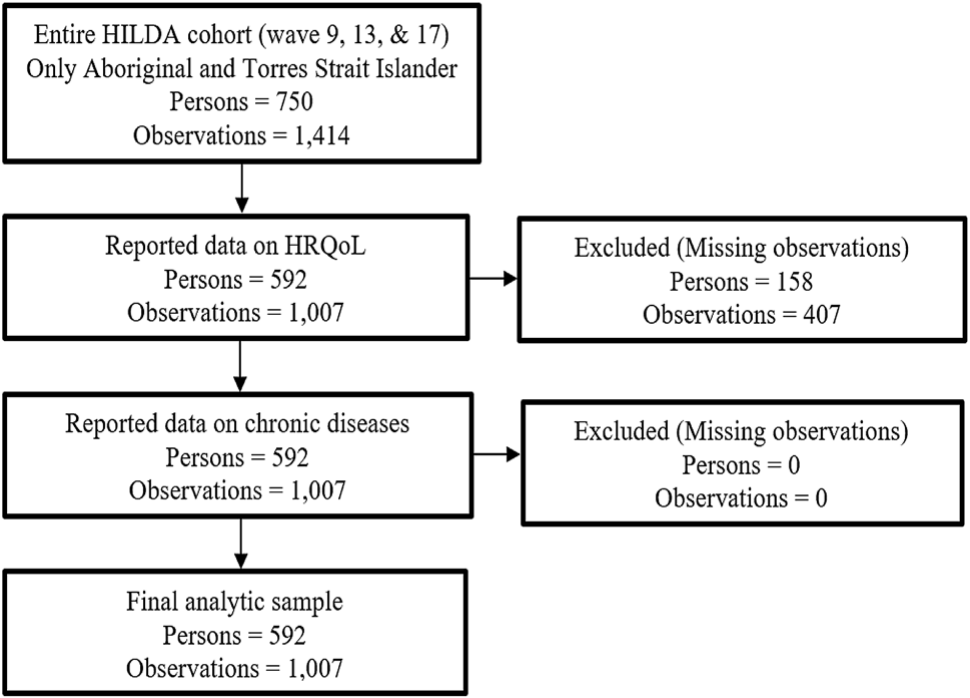
Participants’ flow into the analytic sample and missing data

The subsample used in the current study was constructed from three HILDA Survey waves (9, 13 and 17) covering years 2009, 2013 and 2017. The analyses were restricted to those three data points because they are the only ones containing the required information on chronic conditions. The criteria for participants to be included in the sample were: (i) being aged 15 years and above; (ii) identifying as Indigenous Australians; and (iii) having valid information on the outcome and main variables of interest. Applying this inclusion criteria yielded an unbalanced panel consisting of 1,007 observations from 592 Indigenous Australians. Figure 1 describes the sample selection procedure and missing-data analysis.

### Outcome variable

HRQoL is the primary outcome variable and was approximated using the 36-item Short-Form Health Survey (SF-36). The SF-36 is the most extensively used and accepted health scale to assess individuals’ physical and mental functioning through a standard questionnaire [28]. The questionnaire includes 36 items measuring eight different health dimensions: physical functioning (PF), role physical functioning (RP), role emotional functioning (RE), social functioning (SF), mental health (MH), vitality (VT), bodily pain (BP), and general health (GH). The theoretical range for each dimension of the SF-36 ranges between 0 (worst health) and 100 (best possible health).

Two summary measures are typically calculated using the SF-36 data: the physical-component summary (PCS) and the mental-component summary (MCS) [29]. Four subscales (PF, RP, BP and GH) were combined to generate the PCS and the remaining four subscales (RE, SF, MH and VT) were combined to generate the MCS. The PCS and MCS were standardised by linear z-score transformations, where the mean and deviation were set to 50 and 10, respectively. Both PCS and MCS scores have theoretical ranges spanning from 4.54 to 76.09, and from − 1.21 to 76.19, respectively, with higher scores denoting better health states [30].

Another important HRQoL measure that can be derived from the SF-36 is the health-state utility index, commonly known as the Short-Form Six-Dimension (SF-6D) [31]. The SF-6D is a generic preference-based instrument to measure HRQoL of an individual. It uses information from six SF-36 subscales (PF, RP, RE, SF, VT and BP), and its theoretical range lies between 0.29 (worst health) and 1 (full health) [31]. This index is used as a global measure of HRQoL.

### Exposure variable

Participants self-reported data were used to measure the key exposure variable in this study capturing the experience of no chronic condition, a single chronic condition, or multiple co-occurring chronic conditions (i.e., multimorbidity). HILDA Survey respondents were asked: ‘Have you ever been told by a medical practitioner that you have been diagnosed with a serious illness or medical condition?’, with a list of 11 conditions being presented to them in a showcard to choose from (hypertension, heart disease, type 1 diabetes, type 2 diabetes, chronic bronchitis/emphysema, cancer, asthma, arthritis/osteoporosis, anxiety/depression, other mental health conditions, and circulatory disease). As in previous studies, the exposure variable distinguished three groups of participants: (i) participants experiencing no morbidity, (ii) participants experiencing a single chronic condition (i.e., those reporting only one chronic condition), and (iii) multimorbidity (i.e., those reporting more than one condition) [22, 23, 32].

### Other covariates

Following the existing literature [7, 11, 33–36], several individual-level socio-economic, demographic, lifestyle, and health-related characteristics were included in the multivariable analyses as control variables. The following socio-demographic variables were considered: age (15–29, 30–44, 45–59, and ≥ 60 years), partnership status (single and partnered), highest education level attained (year 12 and below, certificate courses, and university degrees), annual household income (lowest quintile [poorest] to 5 highest quintile [richest], employment status (employed, and unemployed or not in the labour force [NILF]) and area of residence (major city and regional or remote). Smoking status (never smoked, ex-smoker, and current smoker), alcohol drinking (never drunk, used to drink, and currently drinks), and physical activity (less than the recommended level and at recommended level) were included to capture lifestyle factors. Body Mass Index (BMI) was included as a health-related factor (underweight, healthy weight, overweight, and obese).

### Estimation strategy

The analyses begin by summarising the characteristics of the study sample using frequencies, means, standard deviations (SD) and/or percentages at the baseline and final waves, and across all waves pooled. They also summarise the participants’ SF-36 component summary scores, SF-6D score, and SF-36 sub-scale scores according to their multimorbidity status.

We hypothesised that presence of multimorbidity would negatively affect Indigenous Australians’ HRQoL. To test this hypothesis, we employed (symmetric) fixed-effects panel regression models. This technique examines how within-person, over-time changes in multimorbidity status affect within-person, over-time changes in HRQoL. The model fitted here takes the following form:

1 $$HRQoL_{it} - \overline{{HRQoL_{i} }} = \beta (M_{it} - \overline{{M_{i} }} ) + \gamma (X_{it} - \overline{{X_{i} }} ) + \left( {\varepsilon_{it} - \overline{{\varepsilon_{i} }} } \right)$$

In Eq. (1), subscripts *i* and *t* refer to individual and time, respectively; ${HRQoL}_{it}$ represents one of the 11 measures of HRQoL considered; *M* is the main exposure variable capturing respondents’ multimorbidity status; *X* is a vector of control variables; $\beta$ and $\gamma$ are vectors of model coefficients to be estimated; and ${\varepsilon }_{it}$ is the error term.

These (symmetric) fixed-effects regression models use the repeated observations from the same respondents collected at different time-points to estimate how individuals’ changes in multimorbidity status are associated with deviations in their usual HRQoL over time. In doing so, the models implicitly adjust for all time-invariant unobserved factors that could potentially confound the association of interest (e.g., unmeasured lifestyle factors or genetic predispositions). As a result, (symmetric) fixed-effects panel regression models yield estimates which are less affected by omitted-variable bias than traditional cross-sectional regression models.

The model results were reported as adjusted, unstandardized regression coefficients (βs) with 95% confidence intervals (CIs). We also performed Wald tests to ascertain whether the effect of multimorbidity on HRQoL differs from the effect of having a single chronic condition. P-values of < 0.001, < 0.01 and < 0.05 were set to determine the statistical significance of the observed associations. The statistical analyses were executed using Stata 17 software (StataCorp, College Station, Tx, USA).

## Results

### Descriptive analyses

Table 1 shows the socio-economic, demographic, lifestyle, and health-related characteristics of the study sample (n_individuals_ = 592; n_observations_ = 1007). In the pooled data, nearly half (49%) of the participants were between the ages of 15–29 years, 58% were female, and 53% were single. Only 9% of those surveyed had university degrees, 49% were employed, 46% lived in major cities, 40% were current smokers, 76% consumed alcohol, 71% did not perform the recommended level of physical activity, and 30% were obese.

**Table 1:** Summary statistics: socio-economic and demographic, lifestyle, and health-related characteristics

Characteristics	Baseline wave (2009)		Final wave (2017)		Pooled data (2009, 2013 & 2017)	
	n	%	n	%	n	%
Socio-economic, and demographic characteristics						
Age group						
15–29 years	105	46.05	199	48.66	492	48.86
30–44 years	59	25.88	100	24.45	253	25.12
45–59 years	47	20.61	76	18.58	185	18.37
≥ 60 years	17	7.46	34	8.31	77	7.65
Sex						
Male	88	38.60	175	42.79	425	42.20
Female	140	61.40	234	57.21	582	57.80
Marital status						
Single	127	55.70	201	49.14	531	52.73
Partnered	101	44.30	208	50.86	476	47.27
Highest education level attained						
Year 12 and below	156	68.42	227	55.50	602	59.78
Certificate course	54	23.68	144	35.21	312	30.98
University degree	18	7.89	38	9.29	93	9.24
Annual household income						
Lowest quintile (Poorest)	46	20.18	82	20.05	202	20.06
Second quintile	46	20.18	82	20.05	201	19.96
Middle quintile	45	19.74	83	20.29	202	20.06
Fourth quintile	46	20.18	81	19.80	201	19.96
Highest quintile (Richest)	45	19.74	81	19.80	201	19.96
Labour-market status						
Employed	116	50.88	196	47.92	490	48.66
Unemployed or NILF	112	49.12	213	52.08	517	51.34
Area of residence						
Major city	105	46.05	180	44.01	463	45.98
Regional/Remote area	123	53.95	229	55.99	544	54.02
Lifestyle factors						
Smoking status						
Never smoked	81	35.53	145	35.45	362	35.95
Ex-smoker	51	22.37	100	24.45	240	23.83
Current smoker	96	42.11	164	40.10	405	40.22
Alcohol drinking						
Never drunk	17	7.46	45	11.1	97	9.63
Used to drink	26	11.40	72	17.60	149	14.80
Currently drinks	185	81.14	292	71.39	761	75.57
Physical exercise						
Less than the recommended level	158	69.30	300	73.35	716	71.10
Recommended level	70	30.70	109	26.65	291	28.90
Health-related factors						
BMI category						
Underweight	37	16.23	60	14.67	153	15.19
Healthy weight	69	30.26	99	24.21	268	26.61
Overweight	65	28.51	112	27.38	280	27.81
Obese	57	25.00	138	33.74	306	30.39

In the pooled analyses, a total of 1,007 person-year observations from 592 unique persons were included

We equivalised annual household income using the OECD-modified equivalence scale and then categorised into quintiles

*NILF* Not in the labour force. *BMI* Body Mass Index

Table 2 shows the distribution of HRQoL scores, as well as multimorbidity, among the analytic sample. The mean PCS, MCS, and SF-6D scores of the study participants were 48.80, 45.81, and 0.73, respectively. The average score on four of the SF-36’s eight dimensions were: MH (68.78), VT (56.70), BP (70.94), and GH (62.82). In the pooled data, approximately 52% of respondents were free of chronic conditions, about 27% had a single chronic condition, and about a fifth (21%) experienced multimorbidity.

**Table 2:** Summary statistics: subjective health scores, and chronic conditions

Variables	Baseline wave (2009)		Final wave (2017)		Pooled data (2009, 2013, & 2017)	
	n	Mean (SD)	n	Mean (SD)	n	Mean (SD)
SF-36 domain scores						
Physical functioning	228	83.09 (24.23)	409	78.39 (26.31)	1007	80.03 (25.44)
Role physical	228	76.31 (38.32)	409	77.56 (35.79)	1007	77.77 (36.60)
Role emotional	228	80.26 (36.15)	409	77.26 (36.92)	1007	78.33 (36.58)
Social functioning	228	76.75 (26.80)	409	75.45 (25.56)	1007	76.30 (25.58)
Mental health	228	71.59 (18.71)	409	67.04 (19.30)	1007	68.78 (19.28)
Vitality	228	59.09 (21.48)	409	55.26 (20.09)	1007	56.70 (20.75)
Bodily pain	228	74.03 (24.87)	409	69.15 (25.68)	1007	70.94 (25.65)
General health	228	65.46 (21.97)	409	60.71 (21.62)	1007	62.82 (21.81)
SF-36 component summary scores						
PCS	228	49.39 (10.56)	409	48.33 (10.17)	1007	48.80 (10.26)
MCS	228	46.87 (11.61)	409	45.11 (11.60)	1007	45.81 (11.58)
SF-6D	228	0.75 (0.13)	409	0.72 (0.13)	1007	0.73 (0.13)
Number of chronic conditions (% observations)						
0 (No morbidity)	127	55.7	198	48.41	522	51.84
1 (Single chronic condition)	65	28.51	115	28.12	271	26.91
≥ 2 (Multimorbidity)	36	15.79	96	23.47	214	21.25

In the pooled analyses, a total of 1007 person-year observations from 592 unique persons were included

*PCS* Physical component summary, *MCS* Mental component summary, *SF-6D* Short-Form Six-Dimension health utility index

**Fig. 2:**
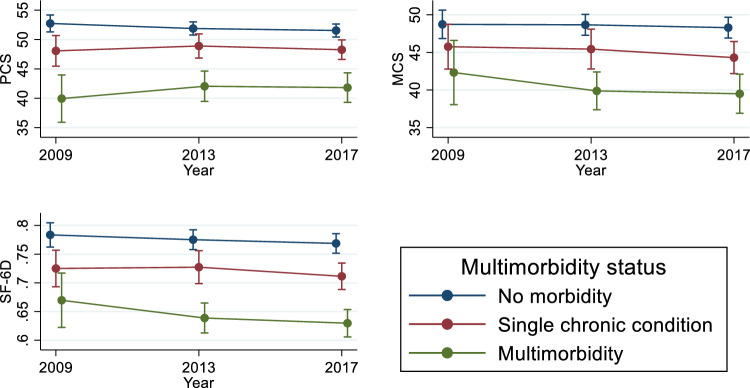
Mean PCS, MCS, and SF-6D utility score by state of chronic conditions. *Notes*: 1. Mean values with corresponding 95% CI. 2. PCS = Physical Component Summary, MCS = Mental Component Summary, and SF-6D = Short-Form Six-Dimension health utility index

Figure 2 illustrates the PCS scores, MCS scores, and SF-6D utility ratings by the values of the (multi)morbidity exposure variable. The results indicate that, across survey waves, Indigenous Australians experiencing multimorbidity exhibited lower PCS, MCS, and SF-6D scores than all other groups. For example, in wave 17 (2017), their average PCS, MCS, and SF-6D scores were 41.81, 39.50, and 0.63, compared to 51.54, 48.29, and 0.77 amongst individuals with no chronic condition, and to 48.26, 44.31, and 0.71 for individuals with single chronic condition.

**Fig. 3:**
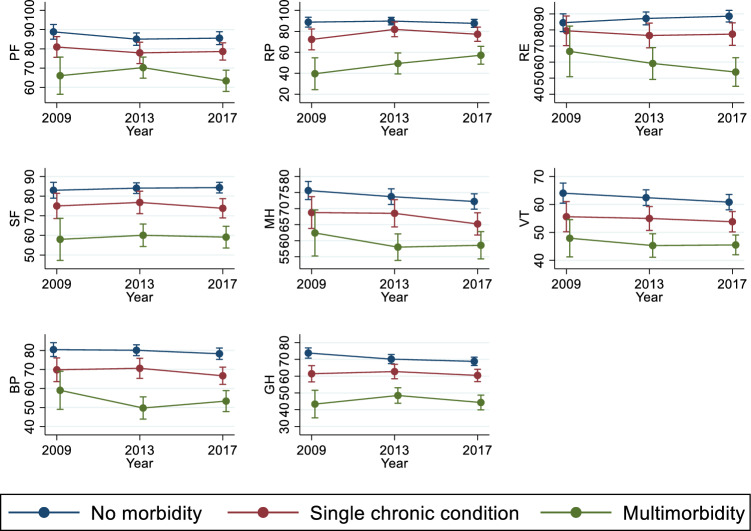
Mean SF-36 sub-scale scores by state of chronic conditions. *Note*: 1. Mean values with corresponding 95% CI. 2. PF = Physical Functioning, RP = Role Physical, RE = Role Emotional, SF = Social Functioning, MH = Mental Health, VT = Vitality, BP = Bodily Pain, and GH = General Health

Figure 3 depicts the mean score of the SF-36’s eight dimensions by the (multi) morbidity exposure variable. As expected, Indigenous Australians with multimorbidity exhibited significantly lower scores in all dimensions of the SF-36 than those with a single chronic condition or no chronic condition. For example, in 2017, the mean PF, RP, RE, SF, MH, VT, BP and GH scores among the participants with multimorbidity (63.37, 57.20, 53.82, 59.11, 58.57, 45.52, 53.35, and 44.30) were substantially lower than those of their counterparts with no chronic condition (85.56, 87.63, 88.55, 84.34, 72.22, 60.83, 78.25, and 68.82) or only one chronic condition (78.62, 77.25, 77.39, 73.80, 65.22, 53.83, 66.67, and 60.46).

Table 3 shows the transition rates (moving from one state to another) for the variable capturing chronic conditions. The rows display the values at the initial timepoint, and the columns show the values at following timepoints. The table shows that 73.78% (166 observations), 48.15% (52  observations), 79.27% (65 observations) of the sample with no chronic condition, single chronic condition, and multimorbidity, respectively, at the baseline remained in the same state in the following timepoints. The table also shows that among the adults with no morbidity at the baseline 19.56% and 6.67% acquired single chronic condition, and multimorbidity, respectively, over the timepoints. Similarly, among the individuals with single chronic condition at the baseline 29.63% had moved to a state of no morbidity and 22.22% had developed multimorbidity. Further, the results showed that amongst individuals with multimorbidity 2.44% had transitioned to no morbidity state and 18.29% had moved to a single chronic condition.

**Table 3:** Estimated transition rate for each category of chronic condition (from T to T + 1 + … + n)

Number of chronic conditions	Number of chronic conditions		
	0 (No morbidity), n (%)	1 (Single chronic condition), n (%)	≥ 2 (Multimorbidity), n (%)
0 (No morbidity)	166 (73.78)	44 (19.56)	15 (6.67)
1 (Single chronic condition)	32 (29.63)	52 (48.15)	24 (22.22)
≥ 2 (Multimorbidity)	2 (2.44)	15 (18.29)	65 (79.27)

1. T indicates the timepoint

2. Total number of yearly observations used for calculating the transition rate is 415

### Regression modelling

Table 4 shows abridged results of the fixed-effects panel regression models. The results from Models 1 and 2 indicate that, all else being equal, respondents with had PCS, MCS and SF-6D scores that were ~ 6.5 (β =  − 6.527; *p* < 0.001), ~ 3.8 (*β* =  − 3.765; *p* = 0.018) and ~ 0.08 (β =  − 0.075; *p* < 0.001) units lower when they experienced multimorbidity relative to when they had no chronic conditions. Respondents experiencing a single chronic condition also had statistically significantly lower scores in the PCS (β =  − 2.942; *p* = 0.002), and SF-6D (β =  − 0.036; *p* = 0.006) than when they had no chronic condition, *ceteris paribus*. Wald tests of equality of coefficients were used to test whether the estimated coefficients of multimorbidity on the PCS, MCS and SF-6D were statistically significantly different to those for having a single chronic condition. The results rejected the equality of coefficients hypothesis at the 5% level for all three models. This indicates that the negative health burden of multimorbidity on HRQoL is larger (i.e., more negative) than the negative health burden of having a single chronic condition.

**Table 4:** Abridged results from fixed effects models of HRQoL (MCS, PCS, and SF-6D)

Model	Outcome	Coefficient on single chronic condition (SE), p-value	Coefficient on multimorbidity (SE), p-value	Wald test: β single chronic condition = β multimorbidity, p-value
Model 1	PCS	**– 2.942 (0.961), 0.002**	**– 6.527 (1.579), < 0.001**	**0.0065**
Model 2	MCS	– 0.824 (1.036), 0.452	**– 3.765 (1.590), 0.018**	**0.041**
Model 3	SF-6D	**– 0.036 (0.013), 0.006**	**– 0.075 (0.017), < 0.001**	**0.008**

1. The sample size is 592 individuals and 1007 observations

2. All models were adjusted for age, marital status, highest education level attained, annual household income, labour market status, area of residence, smoking status, alcohol drinking, physical exercise, and BMI

3. Values in bold denote statistically significant coefficients

4. Ref = Reference Category, PCS = Physical Component Summary, MCS = Mental Component Summary, SF-6D = Short-Form Six-Dimension health utility index, β = Coefficient.

5. Cluster-robust standard errors (SE) are reported in parenthesis

The results of the symmetric fixed-effects panel regression models examining the within-individual associations between multimorbidity status, and the different SF-36 subscales are summarised in Table 5. All else being equal and compared to not having a chronic condition, multimorbidity was associated with statistically significantly lower scores on all SF-36 dimensions except role emotional: PF (β =  − 13.40, *p* < 0.001), RP (β =  − 23.05, *p* < 0.001), SF (β =  − 15.88, *p* < 0.001), MH (β =  − 8.72, *p* = 0.001), VT (β =  − 5.80, *p* = 0.02), BP (β =  − 15.81, *p* < 0.001), and GH (β =  − 11.24, *p* < 0.001). Further, respondents showed statistically significantly lower scores in the PF (β =  − 6.57; *p* = 0.01) and BP (β =  − 9.84; < 0.001) scales in those times points in which they had a chronic condition than in those time points in which they had no chronic condition. Wald tests indicated that the estimated effect of experiencing multimorbidity was larger (*p* < 0.05) than the estimated effect of having a single chronic condition on all SF-36 subscales, except for role emotional, vitality, and body pain.

**Table 5:** Abridged results from fixed effects models of HRQoL (dimensions of the SF-36)

Model	Outcome	Coefficient on single chronic condition (SE), p-value	Coefficient on multimorbidity (SE), p-value	Wald test: β single chronic condition = β multimorbidity, p-value
Model 1	PF	**– 6.57 (2.43), 0.01**	**– 13.40 (3.28), < 0.001**	**0.01**
Model 2	RP	– 5.05 (4.43), 0.25	**– 23.05 (6.10), < 0.001**	**0.0004**
Model 3	RE	**–** 4.70 (4.20), 0.26	– 12.25 (6.84), 0.07	0.20
Model 4	SF	– 4.17 (2.39), 0.08	**– 15.88 (3.64), < 0.001**	**0.0005**
Model 5	MH	– 2.83 (1.78), 0.11	**– 8.72 (2.65), 0.001**	**0.01**
Model 6	VT	– 2.03 (1.87), 0.28	**– 5.80 (2.52), 0.02**	0.09
Model 7	BP	**– 9.84 (2.33), < 0.001**	**– 15.81 (3.69), < 0.001**	0.07
Model 8	GH	– 3.34 (1.96), 0.09	**– 11.24 (2.98), < 0.001**	**0.003**

1. The sample size is 592 individuals and 1,007 person-year observations. 2. All models were adjusted for age, marital status, highest education level attained, annual household income, labour market status, area of residence, smoking status, alcohol drinking, physical exercise, and BMI. 3. Values in bold denote statistically significant coefficients. 2. PF = Physical Functioning, RP = Role Physical, RE = Role Emotional, SF = Social Functioning, MH = Mental Health, VT = Vitality, BP = Bodily Pain, GH = General Health, β = Coefficient. 3. Cluster-robust standard errors (SE) are reported in parenthesis

### Sensitivity analysis

We used multiple imputation (MI) technique as part of sensitivity analyses since the results from the logistic regression (Please refer to Table 3 and 4 in the appendix) showed that some observations were missing at random (MAR). Parameter estimates (Co-efficients) and sampling variances (SE) were obtained from 20 imputed datasets. After performing imputation, we found evidence that multimorbidity is negatively associated with HRQoL and no significant change from the baseline results. The results obtained from the MI estimate showed that a respondent PCS, MCS and SF-6D scores were lower when they experienced multimorbidity relative to when they had no chronic conditions. The results also showed that multimorbidity was associated with statistically significantly lower scores on all SF-36 dimensions except role emotional and vitality. However, the magnitudes of the multimorbidity estimates are slightly different for all the measures of HRQoL. The MI estimates are provided in the appendix (Please refer to Table 5 and 6 in the appendix).

## Discussion

### Key findings

This study has provided novel insights into the association between multimorbidity and HRQoL in an at-risk Australian community, specifically Indigenous Australians. To accomplish this, it leveraged generic non-preference (SF-36) and preference-based (SF-6D) instruments to measure HRQoL; three waves of nationally representative panel data covering the 2009–2017 period; and regression models that yielded estimated coefficients robust to time-invariant unobserved confounders. In the HILDA Survey data, approximately 21% of Indigenous Australians experienced multimorbidity and a further 27% experienced a single chronic health condition. These figures are consistent with national estimates indicating that nearly half of Australians (47%) experience at least one chronic condition in 2020–21 [37], and with the results of a recent study estimating the prevalence of multimorbidity among Indigenous Australians at 24.2% [22]. This consistency provides reassurance about the validity of the (multi) morbidity data in the HILDA Survey.

Our main analyses retrieved the estimated effect of multimorbidity on HRQoL using a fixed-effects modelling approach. The model results confirmed that, consistent with expectations, multimorbidity is significantly associated with reduced HRQoL of Indigenous Australians, as approximated by the PCS, MCS, and SF-6D. We found that the adjusted difference in SF-6D utility values when Indigenous people experienced no morbidities and when they experienced a single chronic condition was moderate in magnitude (28% of a SD). However, the adjusted difference in average utility values when Indigenous people experienced no chronic conditions and when they experienced multimorbidity was large (58% of a SD). Therefore, the results of this study not only are statistically significant, but also hold practical significance. The findings align with results reported in previous studies on broader (i.e., non-Indigenous) populations conducted in high- and low-income countries, whereby multimorbidity was found to be inversely associated with heath-utility scores [7, 33, 34]. Existing research on broader populations has also documented lower PCS and MCS scores amongst adults with multimorbidity in countries such as India, Iran, and the US [11, 35, 36].

In addition, our findings evidenced that the health burden of experiencing multiple co-occurring chronic conditions were observable across multiple domains of Indigenous people’s quality of life. Specifically, multimorbidity was found to decrease HRQoL in seven of the eight dimensions that comprise the SF-36. Prior studies on the relationship between multimorbidity and HRQoL have reported similar findings [38–40], but this is the first study to confirm this pattern of results for Indigenous Australians. When comparing the health burden of multimorbidity on different facets of HRQoL, it becomes apparent that multimorbidity exhibit larger negative associations with SF-36 subscales concerning physical health (PF, RP, BP, and GH) compared to mental health-related subscales (SF, MH, RE, and VT). These results are consistent with existing evidence indicating that people with multimorbidity experienced greater health burden on their physical than their mental HRQoL [34]. They also align with the results of a recent meta-analysis of disease accumulation on quality of life, which showed that physical health declined by − 3.3% with each additional chronic condition, while mental health declined by − 1.6% [33].

Further, our findings reveal that the physical domain of HRQoL was significantly impacted by a single chronic condition, whereas both the physical and mental domains of HRQoL were significantly affected by multimorbidity. This is consistent with a prior study where the authors found that a higher number of comorbidities had a greater negative impact on the mental dimensions of health-related quality of life (HRQoL) [41]. In addition, a recent prospective study revealed that multimorbidity is associated with worse mental HRQOL in a dose–response manner [42]. One possible explanation is that having a greater number of chronic conditions increases the likelihood of depression and anxiety (poor mental health). For example, a prospective study discovered a dose–response relationship between the presence of physical multimorbidity and the occurrence of depression [43].

### Strengths, limitations and avenues for further research

This study features several key strengths. First, the analyses relied on nationally representative longitudinal data, which allowed tracking individuals over time and generating population-generalisable estimates. Second, the panel data were leveraged to fit fixed-effects panel regression models, which provide more robust estimates of the relationships of interest than standard cross-sectional regression models. Third, HRQoL was measured using multiple, high-quality and validated instruments based on the SF-36, safeguarding the validity and reliability of the findings and enabling comparisons of the associations between multimorbidity and different dimensions of HRQoL.

Despite these strengths, some study limitations should be acknowledged. First, the HILDA Survey relies on participants to self-report information on their diagnosed chronic conditions, which can lead to reporting biases. Second, the data did not allow for more detailed refinement of the (multi) morbidity measures; for example, by incorporating information on disease severity, disease knowledge, disease duration, and treatment adherence. Third, while the fixed effects models used here rule time-invariant sources of confounding, they do not protect against reverse causation or other possible biases. As such, our results are to be taken as associational rather than causal. Fourth, it is possible that individuals with low levels of HRQoL or with chronic conditions are more prone to panel attrition, which may influence the results.

Our study also points to possible avenues for further research. For example, it may be important to elucidate the pathways through which multimorbidity affects HRQoL. Previous studies have suggested several putative mechanisms. For instance, commonly co-occurring conditions such as arthritis, cardio-vascular disease, and psychological problems can work together to limit physical functioning, self-care capacity, and social adaptability; increase healthcare-service utilisation and treatment costs; and foster workplace absenteeism or presenteeism [44]. Further, reliance on two or more drugs to treat different chronic conditions may produce synergistic, adverse effects that deplete HRQoL [45]. Each of these processes has been argued to be a likely inhibitor of HRQoL [46–48]. Future research aimed at ascertaining the mechanisms responsible for the lower HRQoL of indigenous Australians with multimorbidity is necessary to better tailor preventive and remedial strategies. In this regard, future research could also deploy *asymmetric* fixed-effects regression models, which could help ascertain whether disease onset and disease end are differentially associated with HRQoL.

### Implications for policy and practice

Indigenous Australians experience substantially poorer health outcomes than the overall Australian population, making them an at-risk group in urgent need of health prevention interventions [19]. The findings reported in this study suggest some possible pathways for the design of health strategies aimed at improving Indigenous health. Approximately one-fifth Indigenous Australians experienced multimorbidity, and their mean utility values (unadjusted) were lower than that of their counterparts with no chronic condition and with a single condition in all timepoints. For example, in 2017, mean utility values among Indigenous Australians with multimorbidity, single, and no chronic condition were 0.63, 0.71, and 0.77, respectively. These substantial health burdens of co-occurring chronic conditions on Indigenous Australians’ HRQoL suggest that multimorbidity should be a consideration in strategies aimed at improving Indigenous health.

Enhancing access to early intervention for chronic disease has been prioritised in the National Aboriginal and Torres Strait Islander Health Plan 2021–2031, with the aim of closing the gap in health and wellbeing outcomes between Indigenous and non-Indigenous Australians [49]. Similarly, the Northern Territory Aboriginal Health Plan 2021–2031 highlighted the need to lower the prevalence of chronic disease by targeting preventative measures and utilising a generalist approach to manage multi-morbidity [50]. Our findings suggest that these strategic directions are appropriate actions to improve Indigenous health outcomes. Comprehensive strategies where patients and health-care providers work in tandem to manage multiple co-occurring chronic conditions can make a difference [51]. Given the experiences of racism and prejudice that Aboriginal people encounter when seeking medical care [52], it is imperative that these strategies cater for the needs and values of Indigenous peoples. This may require specialist training aimed on cultural safety and culturally appropriate health practises.

## Conclusion

Indigenous Australians have substantially higher rates of multimorbidity, both in terms of prevalence and incidence. The present study identified that multimorbidity is significantly and negatively associated with Indigenous people’s quality of life, resulting in a greater health burden on their physical than their mental HRQoL. Future studies that consider the utility value associated with Indigenous multimorbidity, informed by the methods proposed in the current study, are warranted – including studies using causal modelling approaches that can verify the associational relationships reported here.

### Supplementary Information

Below is the link to the electronic supplementary material.

Supplementary file1 (DOCX 34 KB)

## Data Availability

The data were obtained from the Melbourne Institute of Applied Economic and Social Research (https://melbourneinstitute.unimelb.edu.au/). Though the information is not openly available, appropriately qualified researchers can access the data after following their protocols and meeting their requirements. Their contact address is Melbourne Institute of Applied Economic and Social Research, the University of Melbourne, VIC 3010, Australia.

## Abbreviations

BMI: Body mass index
HILDA: Household, income and labour dynamics in Australia
HRQoL: Health-related quality of life
PCS: Physical component summary
MCS: Mental component summary
SF-6D: Short-form six-dimension utility index
SF-36: 36-Item short-form health survey
