# Supplementary material for: Multimorbidity and health-related quality of life amongst Indigenous Australians: A longitudinal analysis
Source: Qual Life Res. 2023 Aug 16;33(1):195–206. doi: 10.1007/s11136-023-03500-3 (PMC10784343; doi:10.1007/s11136-023-03500-3)
Supplement: Supplementary file 1 — Supplementary file1 (DOCX 34 KB) [file 11136_2023_3500_MOESM1_ESM.docx]

STROBE Statement—Checklist of items that should be included in reports of ***observational studies***

|  | Item No | Recommendation | Page No |
| --- | --- | --- | --- |
| **Title and abstract** | 1 | (*a*) Indicate the study’s design with a commonly used term in the title or the abstract | 1 |
|  |  | (*b*) Provide in the abstract an informative and balanced summary of what was done and what was found | 1 |
| Introduction | | | |
| Background/rationale | 2 | Explain the scientific background and rationale for the investigation being reported | 2 |
| Objectives | 3 | State specific objectives, including any prespecified hypotheses | 2-3 |
| Methods | | | |
| Study design | 4 | Present key elements of study design early in the paper | 3 |
| Setting | 5 | Describe the setting, locations, and relevant dates, including periods of recruitment, exposure, follow-up, and data collection | 3 |
| Participants | 6 | (*a*) Give the eligibility criteria, and the sources and methods of selection of participants | 4 |
| Variables | 7 | Clearly define all outcomes, exposures, predictors, potential confounders, and effect modifiers. Give diagnostic criteria, if applicable | 4-6 |
| Data sources/ measurement | 8* | For each variable of interest, give sources of data and details of methods of assessment (measurement). Describe comparability of assessment methods if there is more than one group | 3 |
| Bias | 9 | Describe any efforts to address potential sources of bias | N/A |
| Study size | 10 | Explain how the study size was arrived at | 4 |
| Quantitative variables | 11 | Explain how quantitative variables were handled in the analyses. If applicable, describe which groupings were chosen and why | N/A |
| Statistical methods | 12 | (*a*) Describe all statistical methods, including those used to control for confounding | 6 |
|  |  | (*b*) Describe any methods used to examine subgroups and interactions | N/A |
|  |  | (*c*) Explain how missing data were addressed | 4 |
|  |  | (*d*) If applicable, describe analytical methods taking account of sampling strategy | N/A |
|  |  | (*e*) Describe any sensitivity analyses | 13 |
| Results | | | |
| Participants | 13* | (a) Report numbers of individuals at each stage of study—eg numbers potentially eligible, examined for eligibility, confirmed eligible, included in the study, completing follow-up, and analysed | 4 |
|  |  | (b) Give reasons for non-participation at each stage | 3-4 |
|  |  | (c) Consider use of a flow diagram | 4 |
| Descriptive data | 14* | (a) Give characteristics of study participants (eg demographic, clinical, social) and information on exposures and potential confounders | 7-8 |
|  |  | (b) Indicate number of participants with missing data for each variable of interest | 7-8 |
| Outcome data | 15* | Report numbers of outcome events or summary measures | 8-11 |
| Main results | 16 | (*a*) Give unadjusted estimates and, if applicable, confounder-adjusted estimates and their precision (eg, 95% confidence interval). Make clear which confounders were adjusted for and why they were included | 11-13 |
|  |  | (*b*) Report category boundaries when continuous variables were categorized | 11-13 |
|  |  | (*c*) If relevant, consider translating estimates of relative risk into absolute risk for a meaningful time period | N/A |
| Other analyses | 17 | Report other analyses done—eg analyses of subgroups and interactions, and sensitivity analyses | 13 |
| Discussion | | | |
| Key results | 18 | Summarise key results with reference to study objectives | 14-15 |
| Limitations | 19 | Discuss limitations of the study, taking into account sources of potential bias or imprecision. Discuss both direction and magnitude of any potential bias | 15-16 |
| Interpretation | 20 | Give a cautious overall interpretation of results considering objectives, limitations, multiplicity of analyses, results from similar studies, and other relevant evidence | 14-16 |
| Generalisability | 21 | Discuss the generalisability (external validity) of the study results | 16 |
|  | | | |
| Funding | 22 | Give the source of funding and the role of the funders for the present study and, if applicable, for the original study on which the present article is based | 19 |

*Give information separately for exposed and unexposed groups.

**Note:** An Explanation and Elaboration article discusses each checklist item and gives methodological background and published examples of transparent reporting. The STROBE checklist is best used in conjunction with this article (freely available on the Web sites of PLoS Medicine at http://www.plosmedicine.org/, Annals of Internal Medicine at http://www.annals.org/, and Epidemiology at http://www.epidem.com/). Information on the STROBE Initiative is available at www.strobe-statement.org.

**Appendix**

Table 1 Abridged results from fixed effects models of HRQoL (MCS, PCS, and SF-6D)

| **Parameters** | **(Model 1)** | **(Model 2)** | **(Model 3)** |
| --- | --- | --- | --- |
|  | **PCS** | **MCS** | **SF-6D** |
| Hypertension (Ref = No) | -3.53* | -0.60 | -0.05** |
|  | [-6.34 - -0.72] | [-3.63 - 2.42] | [-0.07 - -0.02] |
| Heart disease (Ref = No) | -6.01* | -1.26 | -0.07* |
|  | [-11.65 - -0.37] | [-7.29 - 4.77] | [-0.13 - -0.02] |
| Circulatory disease (Ref = No) | -4.54* | -1.59 | -0.04* |
|  | [-8.89 - -0.19] | [-5.47 - 2.29] | [-0.08 - -0.00] |
| Arthritis (Ref = No) | -2.87 | 3.19* | 0.00 |
|  | [-5.96 - 0.22] | [0.49 - 5.90] | [-0.03 - 0.04] |
| Asthma (Ref = No) | -1.22 | 1.61 | -0.01 |
|  | [-3.67 - 1.24] | [-1.03 - 4.26] | [-0.04 - 0.03] |
| Cancer (Ref = No) | -3.76 | -6.76* | -0.07* |
|  | [-9.61 - 2.09] | [-11.90 - -1.62] | [-0.13 - -0.01] |
| Bronchitis (Ref = No) | 4.89 | 2.37 | 0.06 |
|  | [-1.72 - 11.50] | [-4.54 - 9.27] | [-0.01 - 0.12] |
| Type 1 diabetes (Ref = No) | -0.41 | 6.15* | 0.00 |
|  | [-7.15 - 6.33] | [0.08 - 12.22] | [-0.07 - 0.08] |
| Type 2 diabetes (Ref = No) | -2.97 | -0.06 | -0.01 |
|  | [-6.90 - 0.96] | [-4.71 - 4.59] | [-0.06 - 0.05] |
| Anxiety/Depression (Ref = No) | -1.32 | -3.76* | -0.05** |
|  | [-3.59 - 0.94] | [-6.89 - -0.63] | [-0.07 - -0.02] |
| Other mental health conditions (Ref = No) | -1.78 | -7.10** | -0.06** |
|  | [-6.80 - 3.25] | [-11.85 - -2.35] | [-0.10 - -0.01] |
| Person-year observations | 1,007 | 1,007 | 1,007 |
| Number of Individuals | 592 | 592 | 592 |

1. All models were adjusted for age, marital status, highest education level attained, annual household income, labour market status, area of residence, smoking status, alcohol drinking, physical exercise, and BMI. 2. Ref = Reference Category, PCS = Physical Component Summary, MCS = Mental Component Summary, SF-6D = Short-Form Six-Dimension health utility index. 3. * indicate significance at the 5% level, ** indicate significance at the 1% level, *** indicate significance at the 0.1% level.

Table 2 Abridged results of fixed effects models of HRQoL (dimensions of the SF-36)

| **Parameters** | **Model 1** | **Model 2** | **Model 3** | **Model 4** | **Model 5** | **Model 6** | **Model 7** | **Model 8** |
| --- | --- | --- | --- | --- | --- | --- | --- | --- |
|  | **PF** | **RP** | **RE** | **SF** | **MH** | **VT** | **BP** | **GH** |
| Hypertension (Ref = No) | -8.44* | -2.76 | -0.44 | -6.52 | -0.85 | -5.18* | -7.35 | -7.14 |
|  | [-15.83 - -1.06] | [-15.17 - 9.65] | [-14.05 - 13.18] | [-15.99 - 2.95] | [-4.91 - 3.20] | [-9.94 - -0.43] | [-15.16 - 0.45] | [-14.34 - 0.06] |
| Heart disease (Ref = No) | -20.09* | -12.36 | -8.30 | -12.21 | -4.05 | -6.50 | -11.72** | 0.01 |
|  | [-36.40 - -3.77] | [-36.66 - 11.93] | [-40.68 - 24.08] | [-28.00 - 3.59] | [-16.01 - 7.92] | [-17.65 - 4.66] | [-20.31 - -3.13] | [-8.66 - 8.68] |
| Circulatory disease (Ref = No) | -5.79 | -19.13** | -19.41* | -0.47 | -0.17 | -6.59* | -9.58* | -9.05** |
|  | [-20.18 - 8.60] | [-33.37 - -4.88] | [-35.74 - -3.08] | [-12.34 - 11.39] | [-7.00 - 6.65] | [-12.31 - -0.86] | [-18.53 - -0.62] | [-15.27 - -2.82] |
| Arthritis (Ref = No) | -6.04 | -3.57 | 5.15 | 4.68 | 1.70 | 5.07* | -3.17 | -2.03 |
|  | [-12.18 - 0.10] | [-16.76 - 9.62] | [-8.66 - 18.96] | [-2.62 - 11.99] | [-3.03 - 6.44] | [0.00 - 10.14] | [-12.51 - 6.16] | [-7.15 - 3.09] |
| Asthma (Ref = No) | -0.06 | -1.69 | 2.27 | 4.85* | 0.44 | 2.36 | -5.32 | -0.04 |
|  | [-6.93 - 6.80] | [-13.53 - 10.15] | [-10.84 - 15.39] | [0.52 - 9.18] | [-4.71 - 5.58] | [-2.47 - 7.18] | [-11.38 - 0.74] | [-6.01 - 5.94] |
| Cancer (Ref = No) | -5.97 | -28.24* | -10.62 | -25.83*** | -8.51 | -14.47** | -7.16 | -5.78 |
|  | [-19.56 - 7.63] | [-54.27 - -2.21] | [-34.09 - 12.86] | [-39.71 - -11.95] | [-19.72 - 2.71] | [-23.10 - -5.83] | [-17.43 - 3.11] | [-15.89 - 4.33] |
| Bronchitis (Ref = No) | 9.96* | 36.83* | 5.83 | 8.06 | 6.78 | 9.71* | 1.62 | 0.87 |
|  | [1.13 - 18.79] | [7.62 - 66.03] | [-5.13 - 16.79] | [-13.61 - 29.73] | [-5.97 - 19.53] | [0.61 - 18.81] | [-16.47 - 19.72] | [-9.60 - 11.34] |
| Type 1 diabetes (Ref = No) | 9.64 | 15.55 | 15.51 | 9.52 | 9.21 | 14.31* | -8.27 | -4.95 |
|  | [-8.88 - 28.17] | [-16.36 - 47.46] | [-12.92 - 43.94] | [-3.25 - 22.30] | [-5.52 - 23.93] | [3.25 - 25.37] | [-23.00 - 6.45] | [-17.43 - 7.53] |
| Type 2 diabetes (Ref = No) | -7.57 | -3.66 | 1.12 | -2.60 | -1.73 | -2.43 | -4.16 | -7.23 |
|  | [-17.02 - 1.89] | [-27.79 - 20.46] | [-24.93 - 27.17] | [-13.96 - 8.75] | [-8.18 - 4.71] | [-11.24 - 6.39] | [-14.03 - 5.71] | [-14.47 - 0.00] |
| Anxiety/Depression (Ref = No) | -3.60 | -7.59 | -13.19* | -8.86* | -6.26* | -3.04 | -7.88* | -3.10 |
|  | [-10.01 - 2.80] | [-16.81 - 1.62] | [-25.04 - -1.34] | [-16.01 - -1.72] | [-11.53 - -1.00] | [-7.61 - 1.53] | [-14.82 - -0.93] | [-7.50 - 1.31] |
| Other mental health conditions (Ref = No) | -7.63 | -3.04 | -8.73 | -20.07*** | -12.42** | -8.59 | -8.55 | -13.07** |
|  | [-21.71 - 6.45] | [-20.96 - 14.88] | [-30.05 - 12.60] | [-30.70 - -9.45] | [-21.62 - -3.23] | [-17.75 - 0.56] | [-19.09 - 1.99] | [-22.14 - -4.00] |
| Person-year observations | 1,007 | 1,007 | 1,007 | 1,007 | 1,007 | 1,007 | 1,007 | 1,007 |
| Number of Individuals | 592 | 592 | 592 | 592 | 592 | 592 | 592 | 592 |

1. All models were adjusted for age, marital status, highest education level attained, annual household income, labour market status, area of residence, smoking status, alcohol drinking, physical exercise, and BMI. 2. Ref = Reference Category, PF = Physical Functioning, RP = Role Physical, RE = Role Emotional, SF = Social Functioning, MH = Mental Health, VT = Vitality, BP = Bodily Pain, and GH = General Health. 3. * indicate significance at the 5% level, ** indicate significance at the 1% level, *** indicate significance at the 0.1% level.

Table 3 Missing observation analysis

| **Variable** | **Missing observations** | **Total observations** | **Percent missing** |
| --- | --- | --- | --- |
| SF-6D utility index | 395 | 1,414 | 27.93% |
| Physical functioning | 342 | 1,414 | 24.19% |
| Role physical | 352 | 1,414 | 24.89% |
| Role emotional | 354 | 1,414 | 25.04% |
| Social functioning | 327 | 1,414 | 23.13% |
| Mental health | 340 | 1,414 | 24.05% |
| Vitality | 340 | 1,414 | 24.05% |
| Bodily pain | 332 | 1,414 | 23.48% |
| General health | 354 | 1,414 | 25.04% |
| All chronic conditions | 0 | 1,414 | 0% |
| Age | 0 | 1,414 | 0% |
| Sex | 0 | 1,414 | 0% |
| Marital status | 0 | 1,414 | 0% |
| Highest education level attained | 0 | 1,414 | 0% |
| Annual household income | 0 | 1,414 | 0% |
| Labour market status | 0 | 1,414 | 0% |
| Remoteness | 0 | 1,414 | 0% |
| Smoking status | 343 | 1,414 | 24.26% |
| Alcohol drinking | 345 | 1,414 | 24.40% |
| Physical exercise | 342 | 1,414 | 24.19% |
| BMI | 460 | 1,414 | 32.53% |

Table 4 Logistic regression results to check which types of observations were less likely to enter each wave

| **Parameters** | **Model 1** |
| --- | --- |
|  | **No missing versus missing observations** |
|  | **aOR (95% CI)** |
| **Number of chronic conditions** |  |
| 0 (No morbidity) (ref) |  |
| 1 (Single chronic condition) | 0.85 [0.57 - 1.27] |
| ≥ 2 (Multimorbidity) | 1.14 [0.69 - 1.87] |
| **Age group** |  |
| 15-29 years (ref) |  |
| 30-44 years | 1.39 [0.90 - 2.15] |
| 45-59 years | 1.20 [0.73 - 1.98] |
| ≥ 60 years | 1.74 [0.84 - 3.57] |
| **Marital status** |  |
| Single (ref) |  |
| Partnered | 0.81 [0.57 - 1.16] |
| **Highest education level attained** |  |
| Year 12 and below (ref) |  |
| Certificate course | 0.79 [0.53 - 1.17] |
| University degree | 0.98 [0.44 - 2.19] |
| **Annual household income** |  |
| Lowest quintile (Poorest) | 0.87 [0.46 - 1.66] |
| Second quintile | 0.58 [0.30 - 1.12] |
| Middle quintile | 0.72 [0.38 - 1.36] |
| Fourth quintile | 0.75 [0.41 - 1.37] |
| Highest quintile (Richest) (ref) |  |
| **Labour-market status** |  |
| Employed (ref) |  |
| Unemployed or NILF | 0.96 [0.63 - 1.47] |
| **Area of residence** |  |
| Major city (ref) |  |
| Regional/Remote area | 0.92 [0.64 - 1.30] |
| **Smoking status** |  |
| Never smoked (ref) |  |
| Ex-smoker | 1.15 [0.68 - 1.95] |
| Current smoker | 1.48 [1.00 - 2.20] |
| **Alcohol drinking** |  |
| Never drunk (ref) |  |
| Used to drink | 1.33 [0.69 - 2.58] |
| Currently drinks | 0.99 [0.57 - 1.70] |
| **Physical exercise** |  |
| Less than the recommended level (ref) |  |
| Recommended level | **0.22*** [0.12 - 0.38]** |
| **BMI category** |  |
| Underweight | **0.03*** [0.02 - 0.05]** |
| Healthy weight (ref) |  |
| Overweight | **0.03*** [0.01 - 0.05]** |
| Obese | **0.02*** [0.01 - 0.04]** |
| **Wave** |  |
| Wave 9 (ref) |  |
| Wave 13 | 0.69 [0.45 - 1.06] |
| Wave 17 | **0.62* [0.40 - 0.97]** |
| Person-year observations | 1,414 |
| Number of Individuals | 750 |

1. aOR= Adjusted Odds Ratio; ref = Reference. 2. * indicate significance at the 5% level, ** indicate significance at the 1% level, *** indicate significance at the 0.1% level. 3. We created a variable entitled “Missingness” (dummy variable) based on the missing observations in the primary outcome variable “SF-6D utility index”. 4. Values in bold denote statistically significant coefficients.

Table 5 Abridged results from fixed effects models of HRQoL (MCS, PCS, and SF-6D) after mean imputation

| **Model** | **Outcome** | **Coefficient on single chronic condition (SE), p-value** | **Coefficient on multimorbidity (SE), p-value** |
| --- | --- | --- | --- |
| Model 1 | PCS | –1.421 (1.146), 0.22 | **–4.594 (1.531), 0.01** |
| Model 2 | MCS | –0.795 (1.331), 0.55 | **–4.618 (1.779), 0.01** |
| Model 3 | SF-6D | **–0.036 (0.014), 0.01** | **–0.054 (0.20), 0.01** |

1. The sample size is 750 individuals and 1,414 yearly observations. 2. All models were adjusted for age, marital status, highest education level attained, annual household income, labour market status, area of residence, smoking status, alcohol drinking, physical exercise, and BMI. 3. Values in bold denote statistically significant coefficients. 4. Ref = Reference Category, PCS = Physical Component Summary, MCS = Mental Component Summary, SF-6D = Short-Form Six-Dimension health utility index. 5. Parameter estimates (Co-efficients) and sampling variances (SE) were obtained from 20 imputed datasets.

Table 6 Abridged results from fixed effects models of HRQoL (dimensions of the SF-36) after mean imputation

| **Model** | **Outcome** | **Coefficient on single chronic condition (SE), p-value** | **Coefficient on multimorbidity (SE), p-value** |
| --- | --- | --- | --- |
| Model 1 | PF | –5.28 (3.00), 0.08 | **–8.17 (3.97), 0.04** |
| Model 2 | RP | –3.91 (4.21), 0.35 | **–12.45 (5.77), 0.03** |
| Model 3 | RE | **–**3.83 (4.42), 0.39 | –8.38 (6.13), 0.17 |
| Model 4 | SF | **–7.11 (3.00), 0.02** | **–13.02 (4.04), 0.01** |
| Model 5 | MH | **–4.17 (2.10), 0.05** | **–7.39 (3.19), 0.02** |
| Model 6 | VT | –1.98 (2.26), 0.38 | –4.56 (3.38), 0.18 |
| Model 7 | BP | **–7.96 (2.94), 0.01** | **–9.36 (3.98), 0.02** |
| Model 8 | GH | –4.12 (2.34), 0.08 | **–7.64 (3.43), 0.03** |

1. The sample size is 750 individuals and 1,414 yearly observations. 2. All models were adjusted for age, marital status, highest education level attained, annual household income, labour market status, area of residence, smoking status, alcohol drinking, physical exercise, and BMI. 3. Values in bold denote statistically significant coefficients. 4. PF = Physical Functioning, RP = Role Physical, RE = Role Emotional, SF = Social Functioning, MH = Mental Health, VT = Vitality, BP = Bodily Pain, GH = General Health. 5. Parameter estimates (Co-efficients) and sampling variances (SE) were obtained from 20 imputed datasets.
